# The feasibility of measuring calprotectin from a throat swab as a marker of infections caused by group A streptococcus: a case–control feasibility study

**DOI:** 10.3399/bjgpopen20X101006

**Published:** 2020-01-22

**Authors:** Behnaz Schofield, Clive Gregory, Micaela Gal, David Gillespie, Gurudutt Naik, Alastair Hay, Nick Francis

**Affiliations:** 1 Senior Research Fellow, Faculty of Health and Applied Sciences, University of West of England, Bristol, UK; 2 Research Manager, School of Medicine Neuadd Meirionnydd, University Hospital of Wales, Cardiff, UK; 3 Institute Translation Manager, School of Medicine Neuadd Meirionnydd, University Hospital of Wales, Cardiff, UK; 4 Deputy Director of Infection, Inflammation & Immunity Trials and Senior Research Fellow, Centre for Trials Research Neuadd Meirionnydd, University Hospital of Wales, Cardiff, UK; 5 Honorary Clinical Lecturer, School of Medicine Neuadd Meirionnydd, University Hospital of Wales, Cardiff, UK; 6 Professor of Primary Care, Centre for Academic Primary Care Bristol Medical School: Population Health Science, University of Bristol, Bristol, UK; 7 Professor of Primary Care Research, School of Medicine Neuadd Meirionnydd, University Hospital of Wales, Cardiff, UK

**Keywords:** leukocyte L1 antigen complex, pharyngitis, anti-bacterial agents, group A streptococci, primary health care, calprotectin, sore throat, antibiotics

## Abstract

**Background:**

Most people with sore throat do not benefit from antibiotic treatment, but nearly three-quarters of those presenting in primary care are prescribed antibiotics. A test that is predictive of bacterial infection could help guide antibiotic prescribing. Calprotectin is a biomarker of neutrophilic inflammation, and may be a useful marker of bacterial throat infections.

**Aim:**

To assess the feasibility of measuring calprotectin from throat swabs, and assess whether individuals with sore throats likely to be caused by streptococcal infections have apparently higher throat calprotectin levels than other individuals with sore throat and healthy volunteers.

**Design & setting:**

A proof of concept case–control study was undertaken, which compared primary care patients with sore throats and healthy volunteers.

**Method:**

Baseline characteristics and throat swabs were collected from 30 primary care patients with suspected streptococcal sore throat, and throat swabs were taken from 10 volunteers without sore throat. Calprotectin level determination and rapid antigen streptococcal testing were conducted on the throat swab eluents. Calprotectin levels in the following groups were compared: volunteers without a sore throat; all patients with a sore throat; patients with a sore throat testing either negative or positive for streptococcal antigen; and those with lower and higher scores on clinical prediction rules for streptococcal sore throat.

**Results:**

Calprotectin was detected in all throat swab samples. Mean calprotectin levels were numerically higher in patients with sore throat compared with healthy volunteers, and sore throat patients who had group A streptococci antigen detected compared with those who did not.

**Conclusion:**

Calprotectin can be measured from throat swab samples and levels are consistent with the hypothesis that streptococcal infection leads to higher throat calprotectin levels. This hypothesis will be tested in a larger study.

## How this fits in

Sore throat is a common reason for antibiotics to be prescribed, even though most are caused by viral infections. Clinical prediction rules, rapid antigen detection testing, and throat culture are all used to try and detect streptococcal throat infections, but none have been shown to accurately differentiate those who need antibiotics from those who do not in a timely fashion. Calprotectin is a marker of neutrophilic inflammation and, therefore, could potentially be used to help identify bacterial throat infections. This feasibility study found that calprotectin can be measured from throat swabs and provides some proof of concept evidence that levels are numerically higher in people with throat infections.

## Introduction

Sore throat is a common reason for patients to consult their GP in primary care.^1,2^ Individuals frequently consult because of a belief that antibiotics are needed to treat their infection, and sore throat accounts for nearly a third of all antibiotics prescribed in primary care.^3^ Most sore throats, however, are caused by viral infections,^4^ and a Cochrane review found that overall fewer than 10% of antibiotic prescriptions (number needed to treat = 14.4) given for sore throat benefit patients.^4^ However, some throat infections are caused by streptococcal infections (in particular group A streptococci [GAS]), and these are more likely to benefit from antibiotic treatment.^5^ Streptococcal pharyngitis can, in some cases, be complicated by acute rheumatic fever, acute glomerulonephritis, and invasive disease leading to septicaemia;^6^ however, these complications are rare, and many thousands of patients with sore throat would have to be treated with antibiotics to prevent one complication.^6^ While antibiotic prescription rates for sore throats declined significantly from the 1990s until the turn of the century, they have stabilised in recent years. There is still a wide variation (10th–90th percentile range 45%–78%) in prescribing rates among primary care practices.^7,8^

Widespread antibiotic use contributes to the selective pressure driving the development of antibiotic resistance,^9^ but inadequate treatment of streptococcal sore throat can potentially lead to increased complications.

Culture of throat swab samples can be used to identify GAS, but culture does not differentiate between streptococcal infection and colonisation, it requires about 48 hours for a result, and it needs follow-up appointments, and it is therefore not recommended for routine use in the UK.^10^ The National Institute for Health and Care Excellence (NICE) recommends the use of Centor^11^ or FeverPAIN^12^ scoring systems, but the predictive qualities are only moderate.^13,14^ Modern rapid streptococcal antigen tests can very accurately detect the presence of streptococcal species and are available as point of care tests (POCTs) that can be used in primary care, but they do not differentiate between colonisation and infection, and have not yet been shown to be better than using clinical criteria in healthcare settings.^12^ A recent Medtech innovation briefing by NICE identified the differences in diagnostic accuracy of this test depending on the population tested, but pointed to its value in increasing diagnostic accuracy when used in conjunction with clinical prediction tools.^15^ A POCT that is objective, sensitive, specific, cost-effective, differentiates between colonisation and infection of the throat, and provides timely results would result in a step-change in the management of sore throat.

Calprotectin is an antimicrobial protein present in the cytoplasm of neutrophil granulocytes.^16^ Calprotectin is secreted in body fluids at the site of tissue inflammation during the acute phase of an infection and is easily measured in a laboratory using enzyme-linked immunosorbent assay (ELISA) test kits, but this is an expensive and time-consuming test.^17^ The current validated clinical test for detection of calprotectin is in screening for inflammatory or infective bowel problems and differentiating these from non-inflammatory causes.^18^ Bacterial and fungal infections are known to cause a predominant neutrophilic response compared with other infections.^19,20^ Calprotectin has been studied with success as a marker of infection with potential to differentiate between bacterial and viral causes in gut infections,^21–24^ and also as a diagnostic marker for prosthetic joint infections.^25^ Calprotectin has been measured from throat swabs in a previous physiological study,^26^ but has not been assessed in patients presenting with sore throat.

A phase I, proof of concept study was proposed using calprotectin as a biomarker in diagnosing throat infections. Based on the above, it was hypothesised that calprotectin levels are greater in throat infections of bacterial origins than in those caused by viral pathogens or where the bacteria are present merely as commensals. The project adopts the architecture for diagnostic research proposed by Sackett and Haynes.^27^ This clinical study is at the first level of assessing diagnostic accuracy, as outlined by Knottnerus *et al*.^28^ The aim in a future, longer-term study is to determine whether calprotectin could be used to distinguish between bacterial throat infection, which is likely to be amenable to antibiotic treatment, and other causes of sore throat in individuals identified as being at higher risk of streptococcal infection, through use of a clinical algorithm (FeverPAIN) and/or rapid streptococcal antigen testing. Specifically, the objective in this study was to determine whether calprotectin can be measured from throat swabs taken from individuals with sore throat and whether those with suspected streptococcal sore throat (SSST) have higher throat swab calprotectin levels than healthy volunteers with no sore throat.

## Method

### Recruitment and participants

Thirty patients (aged ≥18 years) presenting to three South Wales general practices with SSST as their main symptom and 10 volunteers without sore throat symptoms (aged ≥18 years) from the staff base at Cardiff University were recruited to the study. Informed consent was obtained from all individual participants included in the study. Patients were approached by their clinician to be recruited to the study, provided with an information sheet, and informed consent was obtained. Clinicians completed a brief case report form providing basic demographic details, features of the current illness including symptom severity scores, FeverPAIN and Centor as well as clinical features of the sore throat (for example, pharyngeal inflammation), and past medical history, and took a throat swab. Participant inclusion and exclusion criteria are indicated in Box 1.

Box 1 **Participant inclusion and exclusion criteria**

**Table:** 

**Participants**	**Inclusion criteria**	**Exclusion criteria**
Patients	Previously healthy adults (aged ≥18 years) Acute illness (duration ≤7 days) Presenting with sore throat as the main symptom and have a FeverPAIN score ≥2	Patients not consenting Inability to obtain throat swabs Treating clinician believes that the most likely cause of the sore throat is something other than a bacterial or viral infection (that is, thrush, trauma, referred pain, gastro-oesophageal reflux disease) Patients who are known or thought to have significant immune compromise (such as those with leukaemia, lymphoma, myeloma, AIDS, asplenia, congenital immunodeficiency, undergoing chemotherapy, or recent corticosteroid therapy). Patients on immunosuppressive therapy (including oral and inhaled steroids) Active haematological malignancy Undergoing treatment for cancer or has a life-limiting illness (for example, end-stage chronic obstructive pulmonary disease [COPD]) Currently pregnant Have been on antibiotics for this illness
Healthy volunteers	1. Healthy adults (aged ≥18 years) who have had no sore throat symptoms for >4 days	Patients not consenting Patients currently taking antibiotics Inability to obtain throat swabs Active haematological malignancy (undergoing treatment for cancer or has a life-limiting illness [for example, end-stage COPD)]) Currently pregnant

### Sampling procedures

Throat swabs were taken using a double-headed swab (Sigma Swab Duo [without transport medium], MWE) following training and provided instructions. Guidance was provided to the clinicians taking the throat swab samples to try to ensure the same technique, including time period, was used. The swabs were posted (with a completed laboratory requisition form and unique patient identifier) to a Cardiff University laboratory via standard post (Royal Mail). On arrival, the swabs were stored at -80**°**C until all swabs were collected. One head was used for rapid streptococcal antigen detection (Alere TestPack+ Plus StrepA) and the other for measuring calprotectin (calprotectin ELISA kit [MRP 8/14, S100A8/A9, DRG Instruments GmbH]). Strep A antibody and calprotectin analyses were conducted on all swabs at the end of the data collection period. The results of the tests were not returned to clinicians or participants.

On completion of the study, unused swab eluent (eluted in each of the respective kit buffers) was stored for future use in the Archie Cochrane Infections Biobank in accordance with its approved quality procedures (REC reference: 15/WA/0368).

### Alere TestPack +plus StrepA test

As the streptococcal antigen test was developed for point-of-care use, a small feasibility study was undertaken to establish if this test could be used on samples that would be transported by mail at ambient temperature and stored frozen at -80**°**C for a period of time. It was assessed whether use of the streptococcal antigen test was valid in samples that had been transported at room temperature by testing spiked samples stored for up to 48 hours at room temperature and frozen for up to 8 weeks. No evidence was found that storage at room temperature or freezing affected test results.

Participant swabs were posted to the laboratory by mail following collection, stored at -80**°**C, and processed as a batch once sample collection was complete, 29 weeks from receipt of the first sample. Swabs were thawed for 30 minutes and processed following manufacturer’s instructions, noting positive and negative test results.

### Calprotectin test

Before testing for calprotectin, the participant swabs were removed from the freezer and one of the two heads removed and thawed. The remaining swab head was returned to the -80°C freezer for subsequent streptococcal antigen testing. The head of the swab was then centrifuged at 100g for 5 minutes. 400 µL of sample buffer from the calprotectin ELISA kit was added to the swab head and vortexed for 5 seconds. The liquid sample was divided into 100 µL aliquots and frozen at -80°C to allow for batched ELISA analysis. A two-site sandwich calprotectin (Serum) ELISA without dilution was used on the first aliquot, absorbance being measured at 450 nm with a FLUOstar Omega microplate reader (BMG LABTECH Ltd, UK) in accordance with manufacturer’s instructions. Samples outside the upper limit of measurement were re-run at dilutions of 1:10, 1:100, and 1:1000. Analysis of the data were carried out as detailed in the manufacturer’s instructions, applying a 4-parameter-algorithm and run using the FLUOstar’s MARS data analysis software (version 3.00 R3).

### Outcome measure

The outcome measure was calprotectin levels as detectable from throat swabs taken from patients with acute sore throat.

### Statistical analysis

As this was a phase I proof of the concept study, a formal sample size calculation was not conducted. Progression was based on a comparison of means between cases of sore throat and the healthy controls.

The characteristics of the cases and controls were summarised using means and standard deviations (SDs), medians and interquartile ranges (IQRs), and frequencies and percentages, as appropriate. For continuous data, minimum and maximum values were also to be reported to highlight the extremities of the samples. The proportion of cases with confirmed GAS antigen was estimated, with a 95% confidence interval (CI) around the estimate also calculated. Calprotectin levels for cases and controls were summarised using means and medians, along with SDs, IQRs, and minimum or maximum values. Calprotectin values were log transformed (natural logarithm) driven by inspection of the distribution to allow for a comparison of values between cases and controls using means and SDs.

The primary analysis involved calculating the difference in mean calprotectin levels between cases and controls, and fitting a 95% CI around this difference. In order to achieve appropriate coverage probability, the calprotectin level was transformed using the natural logarithm transformation (ln).

Exploratory analyses involved calculating descriptive statistics for calprotectin levels (ln transformed) in: (a) cases with GAS versus cases without GAS; (b) cases with GAS versus controls; (c) cases by FeverPAIN score; and (d) cases by Centor score.

## Results

### Recruited participants

Forty participants (comprising 30 adults with SSST recruited from three primary care practices in South Wales and 10 healthy volunteers) were included between 20 October 2016 and 8 May 2017.

Two participants that were included were subsequently found to have not met the inclusion criteria (*n* = 1 was aged <18 years; and the duration of current illness was >7 days for *n* = 1). These participants have been retained in the analysis. Table 1 describes the characteristics of recruited participants.

**Table 1.** Demographic characteristics of recruited participants

	**Cases (*n* = 30**)	**Controls (*n* = 10**)
	***n***	**%**		***n***	**%**	
**Male**	4	13.3		5	50.0	
**Female**	26	86.7		5	50.0	
	**Mean**	**SD**	**Min–max**	**Mean**	**SD**	**Min–max**
**Age, years**	32.3	11.74	17–64	46.8	10.10	29–61

Min–max = minimum to maximum.

### Clinical features of cases

**Figure 1.**
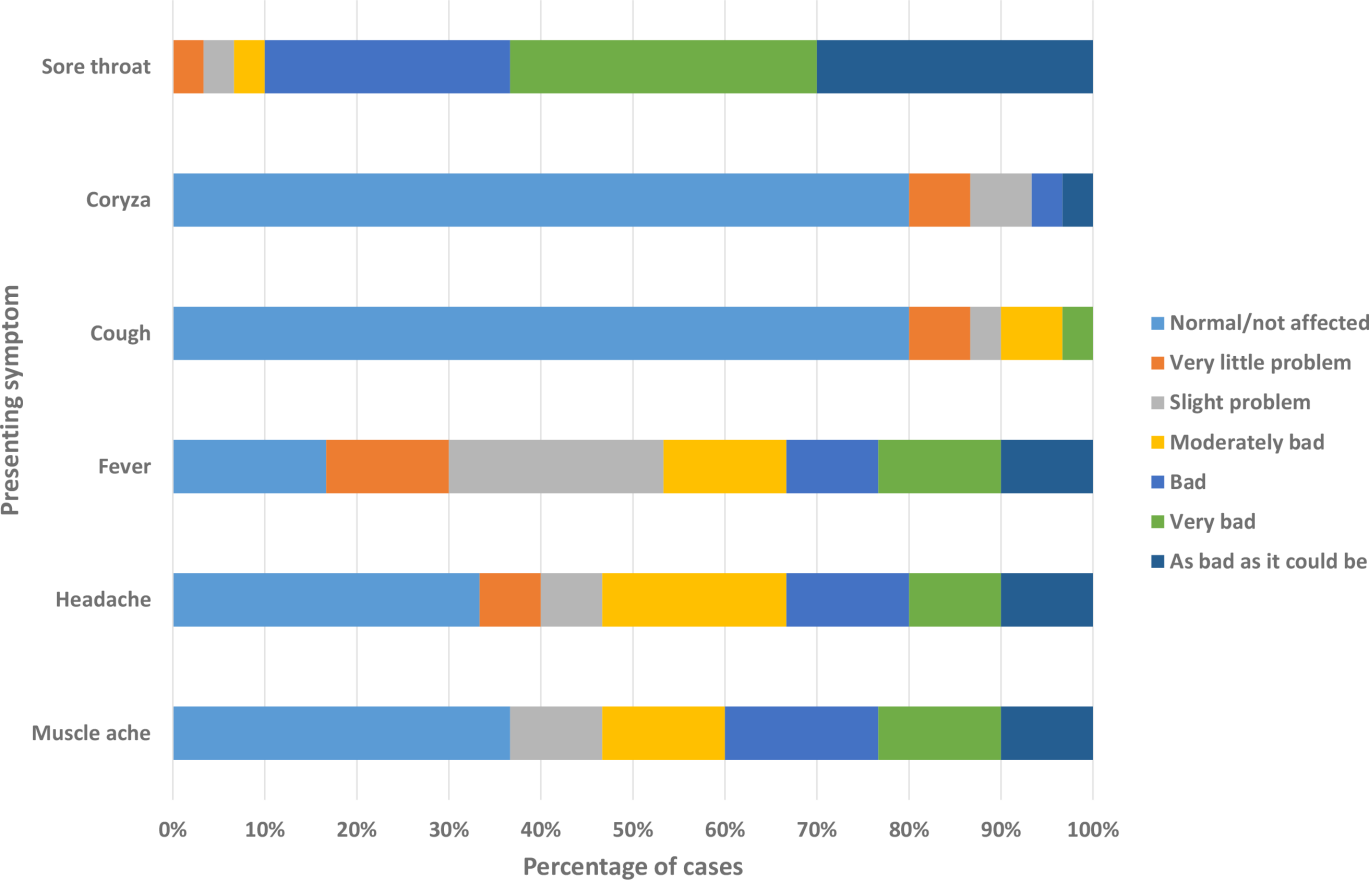
Presenting symptoms and their severity

Cases primarily presented with a sore throat at varying degrees of severity (median 5; IQR 4–6). Fever, headache, and muscle aching were also commonly reported as secondary symptoms, although with lower severity than sore throat (Figure 1). Other clinical features are presented in Table 2.

**Table 2.** Clinical features of cases

	**Cases (*n* = 30**)
			**Median**	**Lower to upper quartile**	**Minimum to maximum**
**Duration of current illness, days**			3	2–3	1–21
**Duration of sore throat, days**			3	2–4	1–7
**FeverPAIN total score**			3	3–4	2–5
**Symptom severity** ^a^	**Sore throat**		5	4–6	1–6
	**Coryza**		0	0–0	0–6
	**Cough**		0	0–0	0–5
	**Fever**		2	1–4	0–6
	**Headache**		3	0–4	0–6
	**Muscle ache**		3	0–4	0–6
**Temperature, ºC** ^b^			37.3	0.70	35.8–39.5
**Centor score**			3	2–3	1–4
			***n***	**%**	
**FeverPAIN total score**		**2**	5	16.7	
		**3**	13	43.3	
		**4**	8	26.7	
		**5**	4	13.3	
**Pharyngeal or tonsillar inflammation**		**None**	2	6.7	
		**Mild**	4	13.3	
		**Moderate**	12	40.0	
		**Severe**	12	40.0	
**Pharyngeal or tonsillar exudate or pus**		**None**	16	53.3	
		**Mild**	4	13.3	
		**Moderate**	5	16.7	
		**Severe**	5	16.7	
**Enlargement of anterior cervical nodes**		**None**	5	16.7	
		**Mild**	7	23.3	
		**Moderate**	15	50.0	
		**Severe**	3	10.0	
**Tenderness of anterior cervical nodes**		**None**	7	23.3	
		**Mild**	3	10.0	
		**Moderate**	10	33.3	
		**Severe**	10	33.3	
**Diabetes**			4	13.3	
**Obesity (BMI >30**)			5	16.7	
**Chronic liver disease**			0	0.0	

BMI = body mass index.

^a^Severity ratings: 1 = normal or not affected; 2 = very little problem; 3 = slight problem; 4 = moderately bad; 5 = bad; 6 = very bad; 7 = as bad as it could be. ^b^Values are mean and standard deviation.

### Streptococcal antigen testing

Samples from eight patients with sore throat (26.7%, 95% CI = 14.2% to 44.5%) tested positive for GAS antigen.

### Calprotectin levels

Calprotectin was present in all samples and the concentration was numerically higher (mean 41816.71, SD 51559.60) for cases with sore throat compared with controls (no sore throat) (mean 5591.51, SD 7877.32). Summary statistics for untransformed and transformed calprotectin levels are given in Table 3. The mean difference in calprotectin levels (ln) was 1.96 (95% CI = 0.90 to 3.02), and the pooled SD (ln) was 1.43.

**Table 3.** Calprotectin values (ng/ml) for cases and controls

	**Untransformed**	**Natural logarithm transformed**
**Cases**(***n* = 30**)	**Controls**(***n* = 10**)	**Cases** **(*n* = 30**)	**Controls**(***n* = 10**)
**Mean (ng/ml**)	41816.71	5591.51	9.80	7.84
**Standard deviation**	51559.60	7877.32	1.40	1.53
**Median**	17045.80	2849.28	9.74	7.91
**Lower to upper quartile**	5983.85 to75835.50	1561.00 to6414.45	8.70 to11.24	7.35 to8.77
**Minimum to maximum**	2057.60 to178115.50	79.95 to26720.00	7.63 to12.09	4.38 to10.19

**Figure 2.**
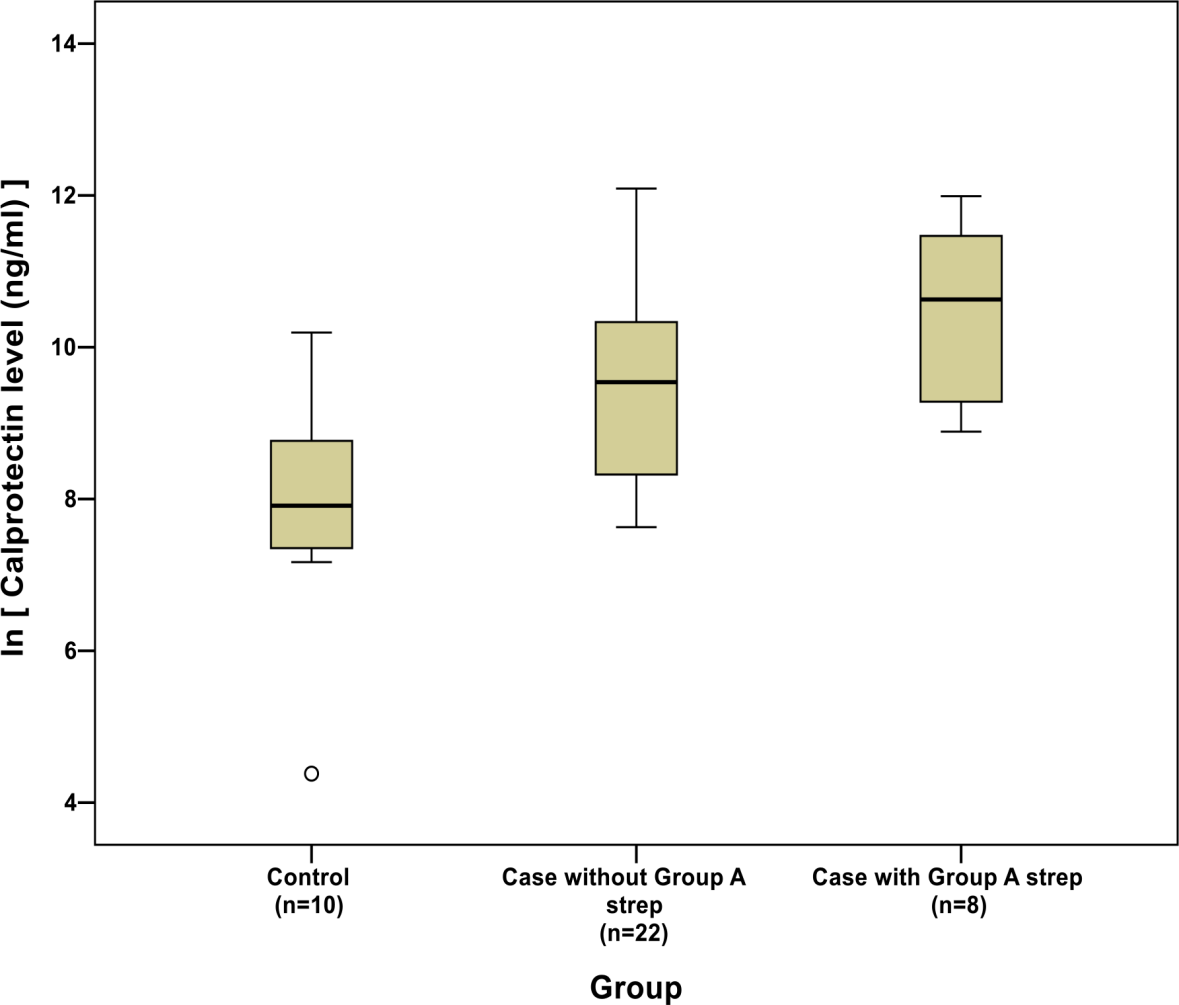
Box plot of calprotectin levels (natural logarithm transformed) for cases (split by group A rapid streptococcus test status) and controls

Cases with detected GAS antigen had numerically higher calprotectin levels than cases without (mean calprotectin level [ln] for cases with GAS = 10.45, SD 1.21; mean for cases without GAS = 9.56, SD 1.41; mean difference = 0.90, 95% CI = -0.25 to 2.05) (Figure 2).

**Figure 3.**
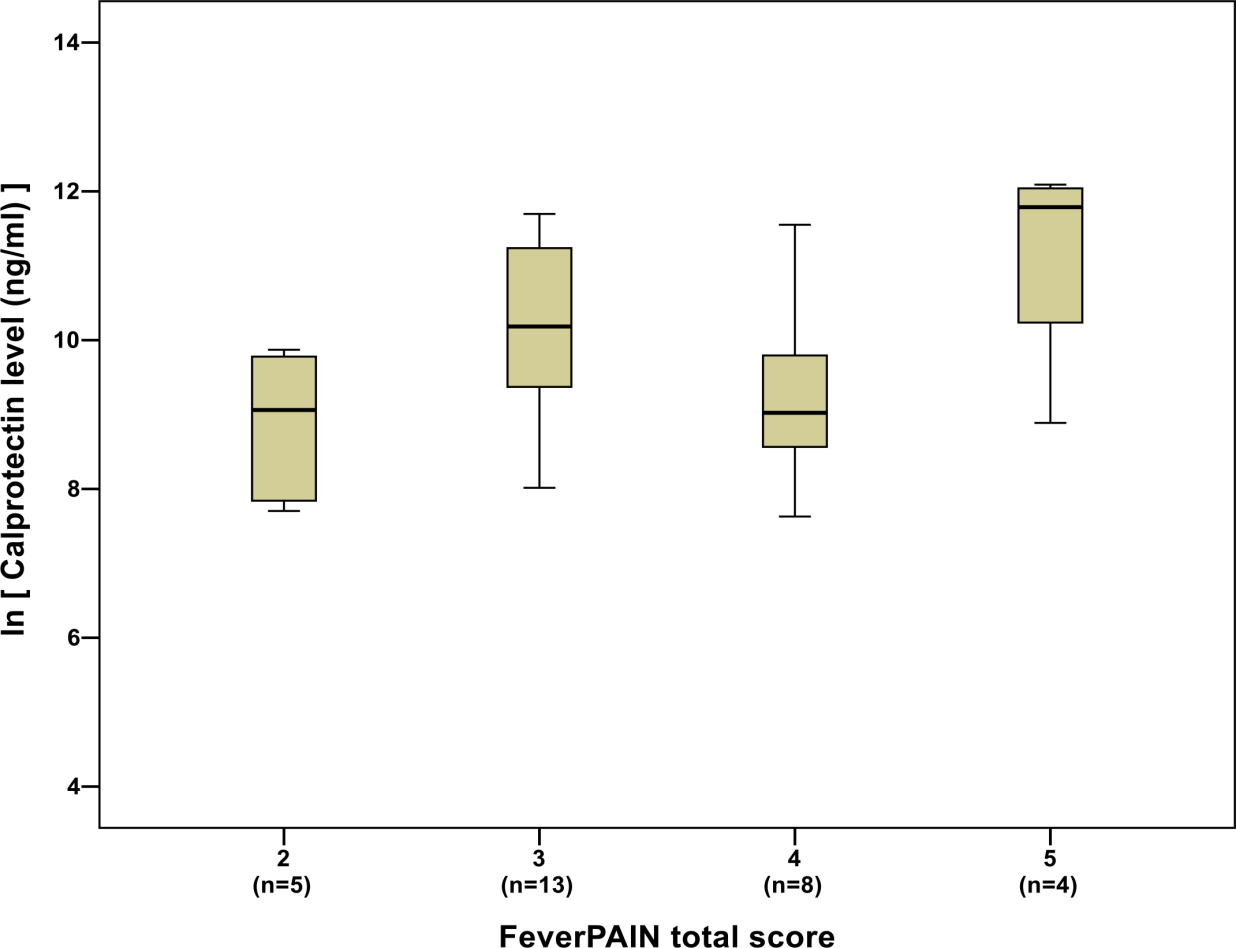
Box plot of calprotectin levels (natural logarithm transformed) by FeverPAIN total score

**Figure 4.**
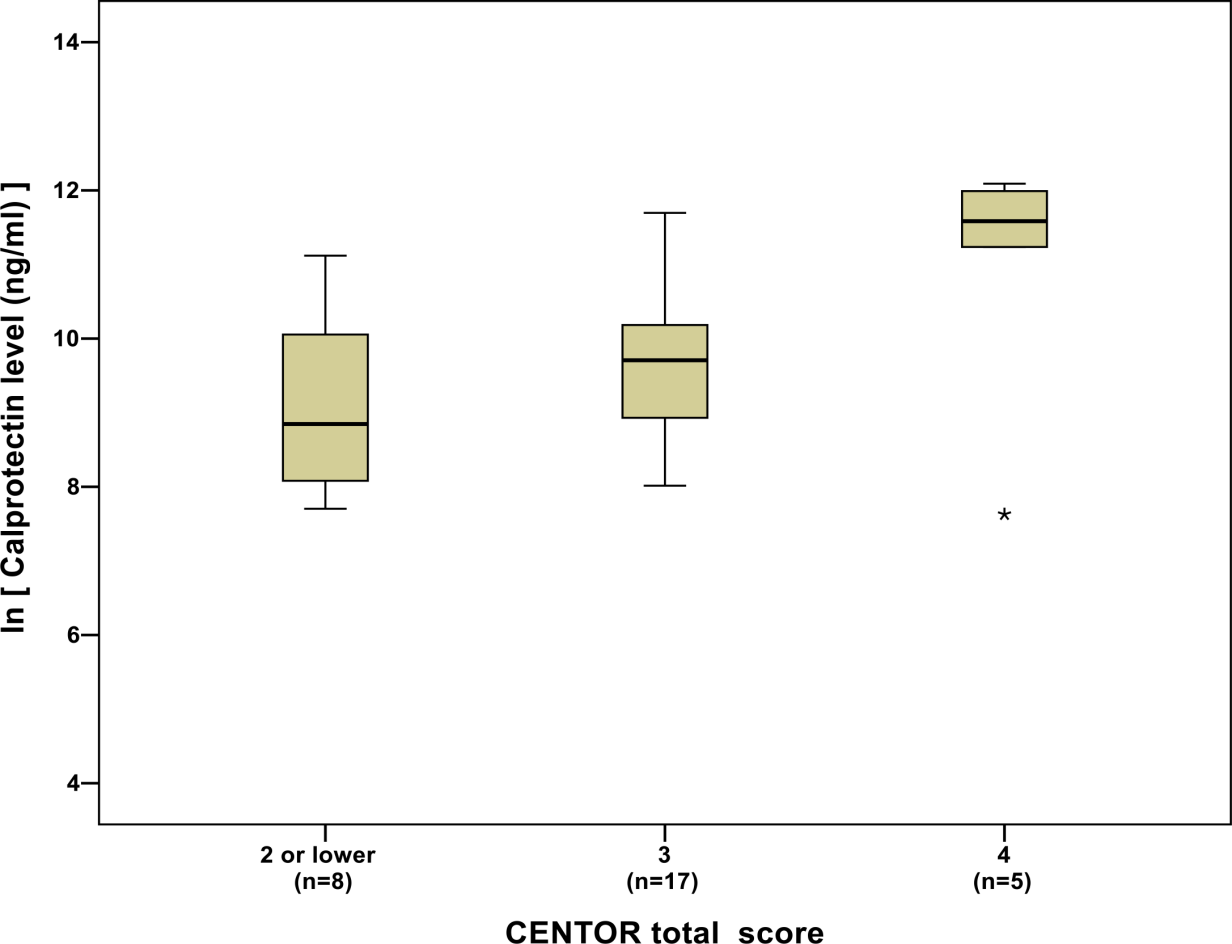
Box plot of calprotectin levels (natural logarithm transformed) by Centor score

While there was no obvious relationship between calprotectin levels and FeverPAIN score (Figure 3), average calprotectin levels increased as Centor scores increased (Figure 4).

## Discussion

### Summary

This is the first study to examine the use of calprotectin levels measured from throat swabs to diagnose bacterial (streptococcal) throat infections. Calprotectin is released by neutrophils and, therefore, is a marker of inflammation. Infection would be expected to cause more inflammation than colonisation, but this would need to be explored in subsequent larger and adequately powered studies. Theoretically, presence of streptococcal antigen coupled with raised local calprotectin levels in patients with sore throat can indicate active acute tissue infection that requires antibiotic treatment. However, this needs to be substantiated in future larger studies. In this proof of concept study, it was demonstrated that calprotectin can be measured from throat swabs, and that calprotectin levels appear to be numerically higher in throat swabs taken from people with suspected streptococcal throat infections (based on clinical scores) than in throat swabs taken from healthy volunteers, and higher in people with sore throat who have GAS antigen detected in their throat than those who do not. The data are also consistent with an association between throat calprotectin levels and clinical predictors of streptococcal sore throat (Centor and FeverPAIN criteria), although these analyses were not powered.

### Strengths and limitations

These findings are consistent with the hypothesis that streptococcal throat infections lead to a neutrophilic inflammatory reaction, and that this can be detected by measuring calprotectin sampled using a throat swab. These findings now need to be confirmed in a larger diagnostic study with patients presenting with acute sore throat in the community.

Potential limitations of the study included small sample size, uncertainty around the effects of sampling technique, lack of matching between cases and controls (for example, sex and age), and lack of a more accurate streptococcal reference standard. A follow-on study will need to use either culture or molecular techniques to detect GAS, and needs to consider detecting streptococci from groups C and G, as these have also been shown to be pathogenic in throat infections. In the next study, only patients with sore throat would be included in order to avoid over-inflating the apparent accuracy of the test. Nevertheless, the objectives of demonstrating the feasibility of sampling and detecting calprotectin from throat swab samples were met, and evidence was provided that an association between throat calprotectin levels and the presence of streptococcal throat infection is possible.

### Comparison with existing literature

Although calprotectin has been measured from throat swabs in a previous physiological study, it has not been assessed in patients presenting with sore throat and, as such, it is not possible to compare the findings of this study with existing literature.

### Implications for research and practice

Calprotectin, or other markers of inflammation, could be developed into a rapid POCT that could be used alongside or instead of clinical prediction rules to guide clinical decision-making in the primary care setting. Such a tool could lead to a revolution in the way that acute sore throat is managed and result in a potential reduction of inappropriate antibiotic prescribing. Funding is now been sought for further studies exploring the utility of host biomarker testing for sore throat. These studies will need to explore the effect of sampling technique as well as how marker detection varies in individuals with no evidence of streptococci in their throat, those who are colonised with streptococci, and those with streptococcal infections.

## References

[1] National Institute of Clinical Excellence (NICE) (2008). Respiratory tract infections — antibiotic prescribing. [NICE Clinical Guideline No 69]. https://www.nice.org.uk/guidance/cg69/evidence/full-guideline-pdf-196853293.

[2] Herath VCK, Carapetis J (2015). Sore throat: is it such a big deal anymore?. J Infect.

[3] Little P, Stuart B, Hobbs FDR (2014). Antibiotic prescription strategies for acute sore throat: a prospective observational cohort study. Lancet Infect Dis.

[4] Spinks A, Glasziou PP DMCB (2013). Antibiotics for sore throat. Cochrane Database Syst Rev.

[5] Shulman ST, Bisno AL, Clegg HW (2012). Clinical practice guideline for the diagnosis and management of group A streptococcal pharyngitis: 2012 update by the infectious diseases Society of America. Clin Infect Dis.

[6] World Health Organization (2005). A review of the technical basis for the control of conditions associated with group A streptococcal infections.

[7] Hawker JI, Smith S, Smith GE (2014). Trends in antibiotic prescribing in primary care for clinical syndromes subject to national recommendations to reduce antibiotic resistance, UK 1995-2011: analysis of a large database of primary care consultations. J Antimicrob Chemother.

[8] Ashworth M, Cox K, Latinovic R (2004). Why has antibiotic prescribing for respiratory illness declined in primary care? A longitudinal study using the general practice research database. J Public Health.

[9] Costelloe C, Metcalfe C, Lovering A (2010). Effect of antibiotic prescribing in primary care on antimicrobial resistance in individual patients: systematic review and meta-analysis. BMJ.

[10] Scottish Intercollegiate Guideline Network (2010). Management of sore throat and indications for tonsillectomy. A national clinical guideline [number 117]. https://www.sign.ac.uk/assets/sign117.pdf.

[11] NICE (2008). Respiratory tract infections (self-limiting): prescribing antibiotics. Clinical guideline [CG69]. https://www.nice.org.uk/guidance/cg69.

[12] Little P, Hobbs FDR, Moore M (2013). Clinical score and rapid antigen detection test to guide antibiotic use for sore throats: randomised controlled trial of prism (primary care streptococcal management. BMJ.

[13] McIsaac WJ, Goel V, To T (2000). The validity of a sore throat score in family practice. CMAJ.

[14] McIsaac WJ, White D, Tannenbaum D (1998). A clinical score to reduce unnecessary antibiotic use in patients with sore throat. CMAJ.

[15] NICE (2018). Point-of-care diagnostic testing in primary care for Strep A infection in sore throat. Medtech Innovation Briefing [MIB145]. https://www.nice.org.uk/advice/mib145.

[16] Dale I, Fagerhol MK, Naesgaard I (1983). Purification and partial characterization of a highly immunogenic human leukocyte protein, the L1 antigen. Eur J Biochem.

[17] Lobatón T, Rodríguez-Moranta F, Lopez A (2013). A new rapid quantitative test for fecal calprotectin predicts endoscopic activity in ulcerative colitis. Inflamm Bowel Dis.

[18] Waugh N, Cummins E, Royle P (2013). Faecal calprotectin testing for differentiating amongst inflammatory and non-inflammatory bowel diseases: systematic review and economic evaluation. Health Technol Assess.

[19] Borregaard N (2010). Neutrophils, from marrow to microbes. Immunity.

[20] Drescher B, Bai F (2013). Neutrophil in viral infections, friend or foe?. Virus Res.

[21] Sýkora J, Siala K, Huml M (2010). Evaluation of faecal calprotectin as a valuable non-invasive marker in distinguishing gut pathogens in young children with acute gastroenteritis. Acta Paediatr.

[22] Duman M, Gencpinar P, Biçmen M (2015). Fecal calprotectin: can be used to distinguish between bacterial and viral gastroenteritis in children?. Am J Emerg Med.

[23] Chen C-C, Huang J-L, Chang C-J (2012). Fecal calprotectin as a correlative marker in clinical severity of infectious diarrhea and usefulness in evaluating bacterial or viral pathogens in children. J Pediatr Gastroenterol Nutr.

[24] Shastri YM, Bergis D, Povse N (2008). Prospective multicenter study evaluating fecal calprotectin in adult acute bacterial diarrhea. Am J Med.

[25] Wouthuyzen-Bakker M, Ploegmakers JJW, Kampinga GA (2017). Synovial calprotectin: a potential biomarker to exclude a prosthetic joint infection. Bone Joint J.

[26] McKay H, Ayers L, Burnett R (2012). Calprotectin as a marker in pharyngeal mucosa and serum, in relation to training in athletes, illness symptoms and cytokines [abstract]. Physiology 2012, Edinburgh, 2012: Proc Physiol Soc 27. https://www.physoc.org/abstracts/calprotectin-as-a-marker-in-pharyngeal-mucosa-and-serum-in-relation-to-training-in-athletes-illness-symptoms-and-cytokines/.

[27] Sackett DL, Haynes RB (2002). The architecture of diagnostic research. BMJ.

[28] Knottnerus JA, van Weel C, Muris JWM (2002). Evaluation of diagnostic procedures. BMJ.

